# Nucleated red blood cells as a novel biomarker in the diagnosis and prediction of sepsis severity in children

**DOI:** 10.3389/fcimb.2023.1264607

**Published:** 2023-11-01

**Authors:** Hongdong Li, Qianqian Tu, Kun Feng, Jie Cheng, Zhiping Zou, Shaojun Li, Liping Tan

**Affiliations:** ^1^ Department of Emergency, Children’s Hospital of Chongqing Medical University, Chongqing, China; ^2^ Ministry of Education Key Laboratory of Child Development and Disorders, Chongqing, China; ^3^ National Clinical Research Center for Child Health and Disorders, Chongqing, China; ^4^ China International Science and Technology Cooperation Base of Child Development and Critical Disorders, Chongqing, China; ^5^ Chongqing Key Laboratory of Child Infection and Immunity, Chongqing, China; ^6^ Department of Clinical Laboratory, Children’s Hospital of Chongqing Medical University, Chongqing, China; ^7^ Department of Neonatology, Children’s Hospital of Chongqing Medical University, Chongqing, China

**Keywords:** nucleated red blood cell, biomarker, sepsis, prognosis, children

## Abstract

**Introduction:**

Sepsis is a vitally serious disease leading to high mortality. Nucleated red blood cells (NRBCs) are present in some noninfectious diseases, but the relationship between NRBCs and sepsis in children remains unknown. The purpose of this study was to compare the clinical characteristics and outcomes of sepsis with positive NRBCs and negative NRBCs in children, and to further explore whether the count of NRBCs has a relationship with the severity of sepsis.

**Methods:**

We enrolled children with sepsis who were admitted to the Children’s Hospital of Chongqing Medical University between January 2020 and December 2022. The children’s clinical data, laboratory data and outcomes were recorded and analyzed.

**Results:**

One hundred and fifteen children met the inclusion criteria in our study. Compared to negative NRBCs patients, the C-reactive protein, alanine transaminase, urea nitrogen values, mortality rate and length of hospitalization were found to be significantly increased, while platelet counts, and hemoglobin were significantly decreased in sepsis patients with positive NRBC (*P* < 0.05). Receiver operating characteristic (ROC) curves analysis showed that the optimal cutoff value of the NRBC count in the diagnosis of severe sepsis was 3, with a sensitivity of 87.5% and specificity of 94.9%. The area under the ROC curve was 0.877 (95% CI: 0.798-0.957).

**Discussion:**

These findings demonstrated that NRBC count has the potential to be a biomarker for the diagnosis of sepsis in children, especially an NRBC count greater than 3, which may predict the severity and poor prognosis in children suffering from sepsis.

## Introduction

Sepsis is life-threatening organ dysfunction caused by the host’s dysregulated anti-infection response, which is the main cause of death in infected patients (Singer et al., 2016; Rudd et al., 2020). Statistics published in the Lancet in 2020 estimated that there were nearly 50 million incident cases of sepsis worldwide each year, with nearly half of them in infants, children and adolescents (Rudd et al., 2020). A meta-analysis on severe sepsis and septic shock in children found an overall sepsis mortality rate of 22.8% for 2011- 2016 (Tan et al., 2019). Sepsis remains one of the leading causes of death among children worldwide (Kissoon et al., 2017). Notably, studies have shown that sepsis-related deaths in children occur extremely quickly, with more than half of deaths occurring within the first 48 hours after admission and even a quarter of deaths occurring within the first 24 hours after admission (MaChado et al., 2017; Schlapbach et al., 2017; Weiss et al., 2017). Therefore, early diagnosis of sepsis and septic shock in children and timely initiation of effective intervention are the top priorities to reduce mortality and improve prognosis.

Conventional diagnostic biomarkers of childhood sepsis include microbial culture and laboratory parameters, including C-reactive protein (CRP), procalcitonin (PCT), white blood cell count (WBC), etc. However, only approximately half of children in intensive care units with a clinical diagnosis of sepsis were microbial culture positive (Irving et al., 2018). Even with the extended microbiological diagnostics performed in the large-scale European Childhood Life-Threatening Infectious Disease Study (EUCLIDS), no pathogen was found in 41% of children with sepsis (Boeddha et al., 2018). In addition, existing laboratory parameters such as CRP, PCT, and WBC counts have limited specificity and sensitivity for evaluating sepsis in children. Therefore, the development of new early diagnostic biomarkers is of great clinical significance for the diagnosis and treatment of sepsis in children.

Nucleated red blood cells (NRBCs) are immature precursors of red blood cells that are not present in the circulation of healthy children (older than 28 days) and adults (healthy newborns have circulating NRBCs that rapidly disappear within a few weeks after birth) (Bahr et al., 2022). However, certain disease states may cause NRBCs to return to circulation. In recent years, several studies have shown that NRBC counts hold great promise for predicting patient mortality. For example, a retrospective cohort study showed that NRBC peak was significantly associated with mortality in critically ill children (Pedersen et al., 2022). The presence of NRBCs may predict mortality in critically ill ICU-admitted patients and COVID-19 patients (Düz et al., 2023; Noor et al., 2023). However, there are few reports on the association between NRBC counts and sepsis. For example, it has been shown that in patients with surgical sepsis, the mortality rate of NRBC-positive patients was higher than that of NRBC-negative patients, and the mortality difference was particularly prominent in surgical patients with severe sepsis (Desai et al., 2012). Additionally, neonates with early-onset neonatal sepsis (EONS) had higher absolute NRBC counts (Dulay et al., 2008). Importantly, there have been no reports of NRBCs in children with sepsis.

In this study, we aimed to evaluate the early diagnostic performance of NRBC count in children with sepsis and to further explore the potential value of NRBC count in risk stratification and prognostic assessment of children with sepsis by comparing the association between NRBC count and disease severity.

## Materials and methods

### Study population

The study was reviewed and approved by the Institutional Review Board of the Children’s Hospital of Chongqing Medical University (No. 133). Written informed consent was obtained from all the guardians of the participants according to the Declaration of Helsinki. This retrospective study analyzed the clinical data of children with sepsis admitted to the Children’s Hospital of Chongqing Medical University (Chongqing, China) from January 2020 to December 2022. The criteria for diagnosis of sepsis were according to the International Consensus Conference on Pediatric Sepsis: 1) two or more criteria for systemic inflammatory response syndrome; 2) confirmed or suspected invasive infection; and 3) cardiovascular dysfunction, acute respiratory distress syndrome, or two or more organ dysfunctions (Goldstein et al., 2005). In addition, all patients involved in the study were diagnosed sepsis at admission. The exclusion criteria were neonates (defined as children < 28 days of age and a correct gestational age of under 44 weeks); hematologic diseases, including hematological malignancies and other hematological diseases; immunodeficiency disease; and hospital stay <3 days with incomplete clinical data. After blood was taken from patients at admission, automated NRBC counts were performed by a Sysmex XN hematology analyzer (Kobe, Japan) in our clinical laboratory.

### Data collection

The clinical data of all children were obtained from electronic medical records and the medical data platform of the hospital, and the demographic characteristics, use of vasoactive drugs, inpatient days, and prognosis of all children were recorded. Laboratory indicators of the patients were recorded at admission and after treatment for one week: ① Routine blood indexes: WBC, platelet count (PLT), and hemoglobin; ② Biochemical indexes: troponin, albumin, alanine aminotransferase (ALT), aspartate aminotransferase (AST), blood urea nitrogen (BUN), serum creatinine (Scr), potassium, sodium, calcium, glucose (Glu) and lactic acid; ③ Inflammatory indexes: CRP and PCT; ④ Coagulation indexes: international normalized ratio (INR) and activated partial thromboplastin time (APTT); ⑤ Pathogenic bacteria data: microbial culture. The outcomes of patients included improvement, poor and death. “Improvement” means that the patients were recovered from the sepsis and discharged from the hospital. “Poor” outcome means that the patient’s condition was further deteriorated after proper treatment. These patients had serious sequelae or withdrawing treatment at last.

### Statistical analysis

Continuous variables are presented as medians (minimum-maximum) unless otherwise indicated. Differences in categorical variables between the groups were analyzed using the χ^2^ test or Fisher’s exact test. The Mann−Whitney U test or t test was used to compare continuous variables between the groups. To predict the probability of poor prognosis for children with sepsis, the NRBC counts of positive NRBC group were used to construct receiver operating characteristic (ROC) curves, and the area under the curve was determined. The optimal cutoff value for the NRBC count in the context of ROC curve analysis is the maximum of the Youden index. SPSS 26.0 (IBM Corp., Armonk, New York, USA) was used for all statistical analyses and graphing. *P* values less than 0.05 were considered statistically significant.

## Results

### Comparison of demographic characteristics of children with sepsis in the NRBC-positive and NRBC-negative groups

A total of 115 children with sepsis were included in the study, with 77 patients being NRBC positive and 38 patients being NRBC negative. The demographic characteristics of the subjects are listed in **Table 1**. There were no differences in sex, age, weight, course of disease, or basic disease between the positive and negative groups (P >0.05). However, there was a significant difference in surgical history (P <0.001) and source of infection (P <0.05) between the NRBC-positive and NRBC-negative groups. Furthermore, NRBC counts were associated with the severity of sepsis; severe sepsis in the NRBC-positive group was significantly higher than that in the NRBC-negative group (17% and 3%, respectively) (p=0.03), and septic shock in the NRBC-positive group was also significantly higher than that in the NRBC-negative group (18% and 10%, respectively) (p=0.02).

**Table 1:** Demographic characteristics in children with bacterial sepsis.

	NRBC (+) (N=77)	NRBC (-) (N=38)	*P*-value
Age, month	26(1.2-180)	33(2.3-172)	0.29
Sex, n (%)			
Female	43(56)	22(58)	0.33
Male	34(44)	16(42)	0.17
Weight, kilograms	11(4-18)	12(4.5-18.9)	0.22
Course of disease, days	3(1-9)	3.5(2-6)	0.56
Basic disease, n (%)	43(56)	11(29)	0.12
Surgical History, n (%)	32(42)	3(4)	<0.001
Source of infection, n (%)			
Abdomen	17(22)	4(10)	<0.001
Respiratory tract	34(44)	22(58)	0.03
Blood stream	8(10)	1(3)	0.04
Urinary tract	1(1)	8(21)	0.01
Soft tissue	5(7)	0(0)	0.02
Others	12(16)	3(8)	0.004
Classification, n (%)			
Sepsis	50(65)	33(87)	0.25
Severe sepsis	13(17)	1(3)	0.03
Septic shock	14(18)	4(10)	0.02
Positive blood culture	12(16)	1(3)	0.03

NRBC, Nucleated red blood cell.

Categorical variables are expressed as absolute numbers (percentage). Continuous variables are expressed as median (interquartile range).

### Comparison of laboratory indicators of patients between the NRBC-positive and NRBC-negative groups at admission and after treatment

**Figure 1:**
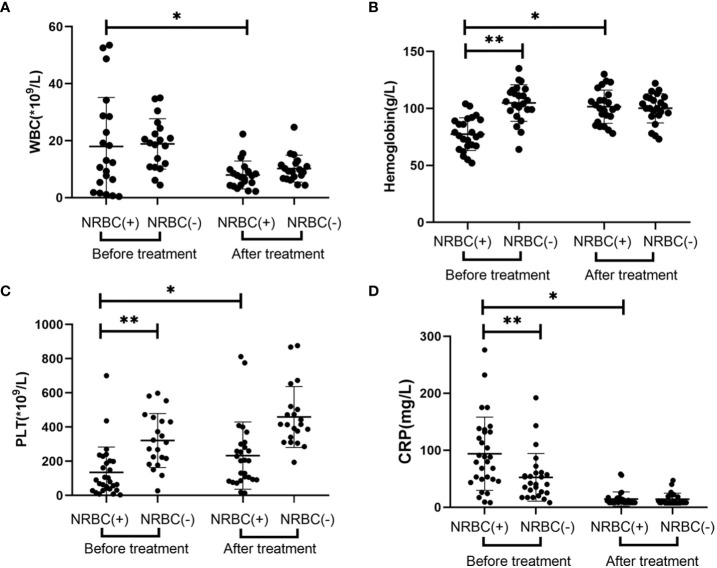
Blood index for septic children with and without nucleated red blood cells (NRBCs) before and after treatment. WBC **(A)**, Hemoglobin **(B)**, PLT **(C)** and CRP **(D)** levels present in the blood of the respective groups are shown. One asterisk denotes a statistically significant difference from the NRBC (+) group after treatment (*P* < 0.05), double asterisks denote a statistically significant difference from the NRBC **(-)** group before treatment (*P* < 0.05). WBC, white blood cell; PLT, platelet; CRP, c-reactive protein.

**Figure 2:**
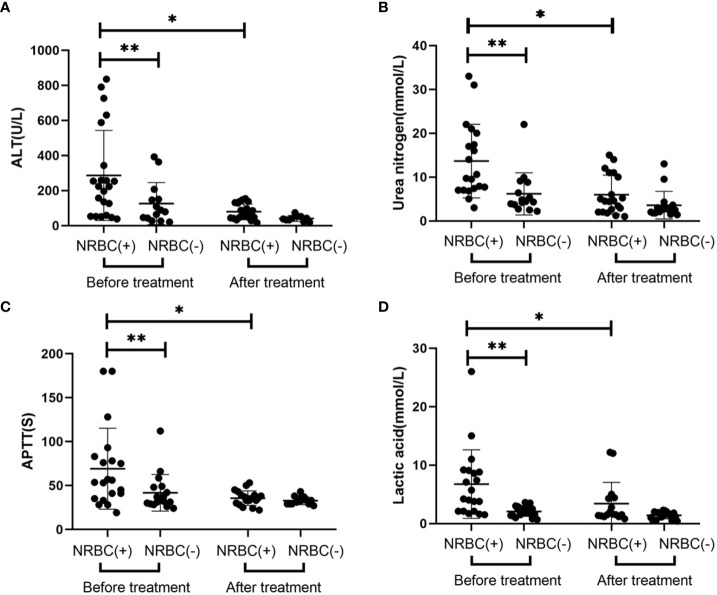
Laboratory values for septic children with and without nucleated red blood cells (NRBCs) before and after treatment. ALT **(A)**, urea nitrogen **(B)**, APTT **(C)** and lactic acid **(D)** levels present in the blood of the respective groups are shown. One asterisk denotes a statistically significant difference from the NRBC (+) group after treatment (*P* < 0.05), double asterisks denote a statistically significant difference from the NRBC **(-)** group before treatment (*P* < 0.05). ALT, alanine aminotransferase; APTT, activated partial thromboplastin time.

In terms of laboratory parameters, the blood indexes were significantly improved, and there were no significant differences in the blood indexes between the two groups after treatment (**Figure 1**). There were statistically significant differences in laboratory indicators between NRBC-positive and NRBC-negative patients at admission. In the blood parameters, platelet count and hemoglobin in the NBRC-positive group were significantly lower than those in the NRBC-negative group, while CRP was higher in the NBRC-positive group (P <0.05) (**Figure 1**). Among the biochemical indexes, the ALT, BUN, lactic acid and APTT levels in the NRBC-positive group were higher than those in the NRBC-negative group (P <0.05) (**Figure 2**). Furthermore, blood culture in NRBC-positive patients had a higher positive rate than that in NRBC-negative patients (P <0.05) (**Table 1**). However, other parameters, such as PCT, troponin, albumin, AST, Scr, potassium, sodium, and calcium, showed no difference between the two groups (data not shown).

### Comparison of severity and prognosis of patients in the NRBC-positive and NRBC-negative groups

**Figure 3:**
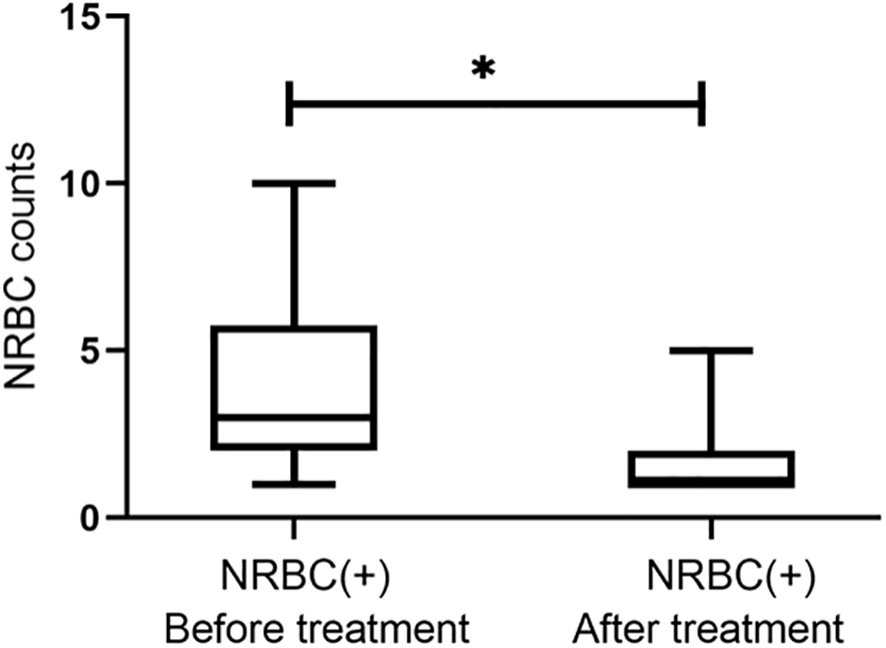
The trend of nucleated red blood cell (NRBC) count for septic children before and after treatment. Asterisk denotes a statistically significant difference from the NRBC (+) group after treatment (*P* < 0.05).

The criteria for treatment of sepsis were also according to the International Consensus Conference on Pediatric Sepsis (Goldstein et al., 2005). NRBC-positive patients were more likely to have transfusion (55/77, 71% vs. 8/38, 21%, respectively) and hemopurification (12/77, 16% vs. 2/38, 5%, respectively) treatments (**Table 2**). The main method of hemopurification for sepsis is hemodiafiltration. Moreover, NRBC-positive patients appeared to have more inpatient days (median 17.6 vs. 10.5, respectively; Mann−Whitney p= 0.03). To further evaluate the role of NRBC count in predicting mortality in children with sepsis, we compared outcomes in the NRBC-positive and NRBC-negative groups. The vast majority of the NRBC-negative patients recovered after treatment (97.4%), while 25.9% of the NRBC-positive patients experienced deterioration or even death after treatment (**Table 2**). Additionally, the counts of NRBC in NRBC-positive children who recovered from diseases significantly decreased after treatment (**Figure 3**). These results indicated that NRBC-positivity may be linked with a poor prognosis of childhood sepsis.

**Table 2:** Treatment and clinical outcomes in children with sepsis.

	NRBC (+) (N=77)	NRBC (-) (N=38)	*P*-value
Surgery during hospitalization, n (%)	13(17)	3(8)	0.31
Respiratory support, n (%)			
Invasive	19(25)	6(16)	0.54
Non-invasive	3(4)	2(5)	0.71
None	55(71)	30(79)	0.23
Transfusion, n (%)	55(71)	8(21)	<0.001
Vasoactive agent, n (%)			
None	62(81)	33(86)	0.64
One	1(1)	1(3)	0.58
Two	5(7)	2(5)	0.42
vThree	8(10)	1(3)	0.12
Four	1(1)	1(3)	0.58
Use of hemopurification, n (%)	12(16)	2(5)	0.04
Hospital stay, days	17.6(10-21.3)	10.5(7.3-19.8)	0.03
Outcomes, n (%)			
Improvement	57(74.1)	37(97.4)	0.04
Poor	15(19.5)	1(2.6)	0.01
Death	5(6.5)	0	0.004

NRBC, Nucleated red blood cell.

Categorical variables are expressed as absolute numbers (percentage). Continuous variables are expressed as median (interquartile range).

### Predictive value of poor prognosis by NRBC count in children with sepsis

**Figure 4:**
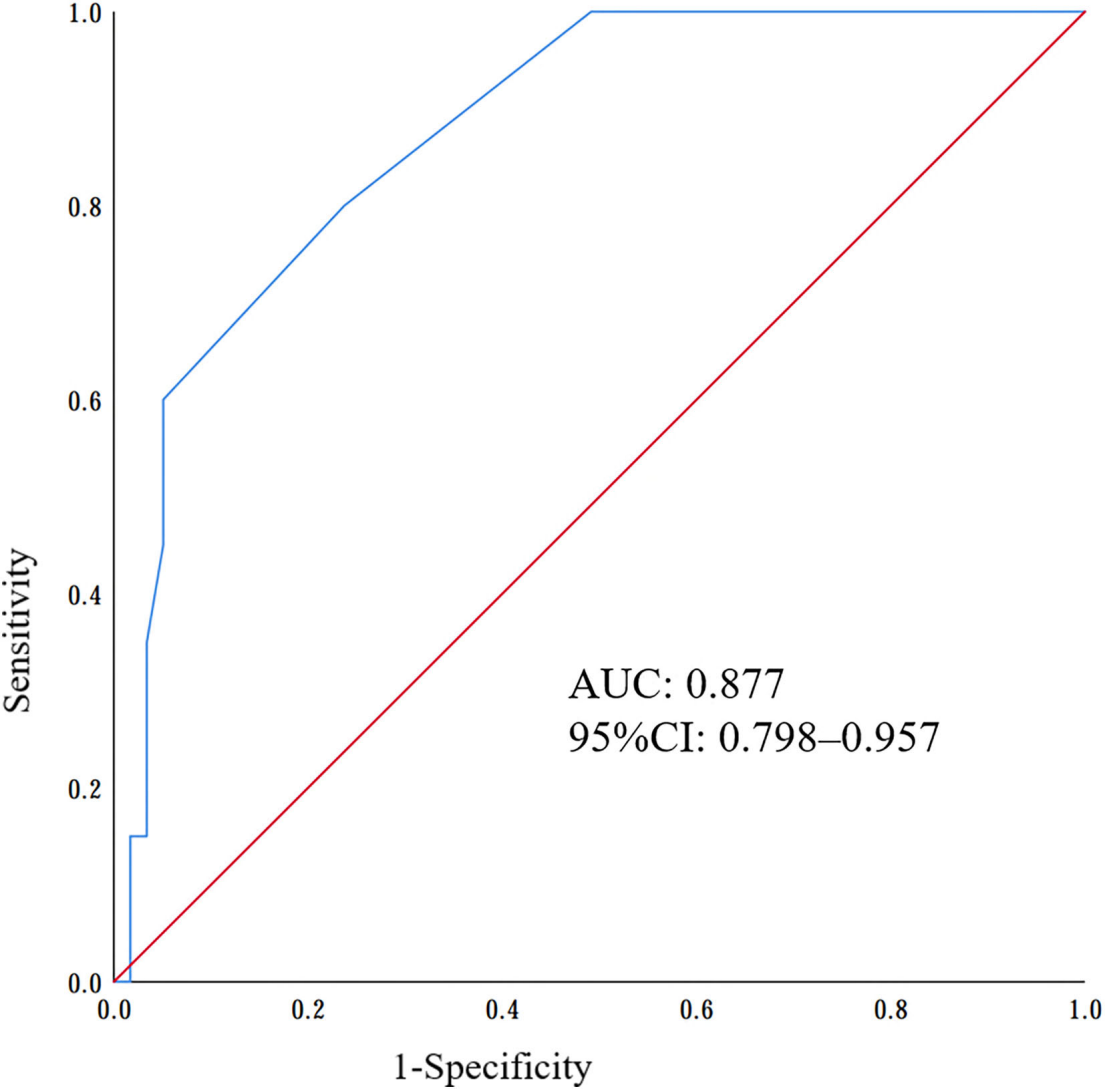
Receiver operating characteristic (ROC) curves of nucleated red blood cells (NRBCs) to predict the poor prognosis of sepsis.

Receiver operating characteristic (ROC) curve analysis confirmed the predictive value of poor prognosis in children with sepsis, and the area under the curve (AUC) of the ROC curve was 0.877 [95% confidence interval (CI) 0.798–0.957] (**Figure 4**). Based on the ROC analysis, the optimal cutoff value for the NRBC count was 3, with a sensitivity of 87.5% and specificity of 94.9% (**Figure 4**).

## Discussion

Sepsis is a disease with high morbidity and mortality (Vincent et al., 2014; de Souza et al., 2021). Statistics showed that in 2017, there were an estimated 48.9 million new cases of sepsis and 11.0 million sepsis-related deaths, representing 19.7% of deaths globally (Rudd et al., 2020). While poor prognosis can be reduced by prompt initiation of sepsis protocols, including fluid resuscitation and antibiotic therapy, the provision of these therapies relies on early recognition of sepsis by clinicians (Evans et al., 2018). This recognition is particularly challenging in children because abnormal vital signs such as fever, tachycardia, and tachypnea are less specific in identifying children in the early stages of septic shock (Scott et al., 2015). Therefore, researchers have turned their attention to the development of diagnostic biomarkers. At present, the most commonly used biomarkers of sepsis in children are WBC, PCT and CRP. CRP is a widely used biomarker of infection. However, CRP levels rise slowly and take 1-2 days to peak during infection, resulting in its low sensitivity in the early stages of the disease (Lelubre et al., 2013). CRP is not appropriate for early diagnosis. As a precursor protein of calcitonin, PCT is a commonly used biomarker for sepsis. In a prospective study of 80 children with suspected sepsis in the pediatric intensive care unit (PICU), PCT levels were superior to CRP levels or WBC counts in diagnosing severe infections (Casado-Flores et al., 2003). However, the diagnostic efficacy of PCT in children at high risk of nonbacterial sepsis remains unclear (Downes et al., 2020). Its high price and limited detection technology lead to a narrow range of application, and it is not suitable for primary hospitals. Therefore, there is an urgent need for routinely available biomarkers to help clinicians identify early childhood sepsis, especially severe sepsis and septic shock in children.

In this study, we found that children with NRBC-positive sepsis had higher disease severity, with longer hospitalization times, higher utilization rates of transfusion and hemopurification, and a higher probability of poor prognosis. In addition, ROC curve analysis showed that NRBC count had the potential to be a biomarker for the early diagnosis of childhood sepsis; in particular, an NRBC count greater than 3 could predict the severity and poor prognosis of childhood sepsis. Notably, there are physiologically circulating NRBCs in healthy newborns, so this cutoff value is not appropriate for early-onset neonatal sepsis (EONS). Furthermore, only one NRBC-negative patient had a poor prognosis (2.6%), suggesting that NRBC-negative status had the potential to be a negative predictor of mortality in children with sepsis. Additionally, a septic animal model using pig showed that NRBCs became significantly elevated after two hours of infection (Tóth et al., 2017).

In other populations, the relationship between circulating NRBC counts and poor outcomes in sepsis has been well described. A study of 68 patients with EONS showed that neonates with EONS had higher NRBC counts (p=0.011), and the NRBC count was directly correlated with IL-6 levels in cord blood *(P* < 0.001) (Dulay et al., 2008). Another retrospective study of adult patients with surgical sepsis showed that mortality was higher in NRBC-positive patients, and the mortality difference was particularly pronounced among surgical patients with severe sepsis (Desai et al., 2012). Except in newborns, NRBC count is generally considered a biomarker of severe stress or pathological erythropoiesis, and systemic inflammation, hypoxia, and massive bleeding can all lead to elevated NRBC count (Constantino and Rivera, 2019). The mechanism of the elevated NRBC count in children with sepsis remains unclear, and we speculate that the elevated NRBC count in children with sepsis may be due to tissue hypoxia caused by hypoperfusion. In our study, NRBC-positive patients had higher serum lactic acid levels, supporting the role of hypoxia in the production and release of NRBCs (Wang et al., 2023). Also, non-statistically significant WBC differences and lower platelet counts mean patients with elevated NRBC are immunosuppressed. Another possibility of the mechanism was that NRBCs seem to have a regulatory function via the induction of IL-10 production by monocytes to suppress a vigorous innate immune reaction, as IL-10 plays a detrimental role in sepsis (Cui et al., 2016; Xie et al., 2021; Zhang et al., 2022).

Sepsis-related immunosuppression is associated with an increased risk of death, and it has been reported that suppression of innate and adaptive immunity within 48 hours of sepsis onset was significantly associated with a longer duration of organ dysfunction (Muszynski et al., 2018). In this study, WBC and platelet counts of NRBC-positive patients were lower than those of NRBC-negative patients, indicating that the immunosuppression of NRBC-positive patients was more severe, which may be one of the reasons for the increased mortality of NRBC-positive patients, but the causal relationship between increased NRBC count and aggravated immunosuppression needs to be further clarified. Furthermore, we observed the NRBC count at admission and monitored the change trend of the NRBC count. The counts of NRBC significantly decreased after treatment in NRBC-positive children who recovered from diseases. A study of 9,690 adult patients admitted to the surgical ICU showed a protective value of NRBC counts returning to 0 compared with NRBC counts never returning to baseline during admission (Shah et al., 2012). Therefore, monitoring changes in NRBC counts may be helpful in observing the disease trajectories of patients.

There are several limitations to our study. First, all cases were from the Children’s Hospital of Chongqing Medical University, leading to relatively small sample sizes, and larger sample sizes may provide more convincing results. Further results of a multicenter study would carry more weightage. Second, the definite mechanism of NRBC is unknown, and further research is needed to explain this question. At last, it was a retrospective study, leading to potential bias when collecting clinical data from our medical charts.

## Conclusions

In summary, this retrospective study suggests that NRBC count is a valuable biomarker for early diagnosis of sepsis in children with superior diagnostic performance, and elevated NRBC count is associated with poor prognosis of sepsis in children, which can guide follow-up treatment and allow for implementation of better intervention protocols in clinical practice to improve prognosis. Notably, NRBC counts are regular components of the analysis procedures on blood analyzers, and the results are available in a short time and without any additional cost. Therefore, the NRBC count is easy to popularize in primary hospitals and is a relatively ideal biomarker in clinical practice.

## Data availability statement

The original contributions presented in the study are included in the article/supplementary material. Further inquiries can be directed to the corresponding author.

## Ethics statement

The studies involving humans were approved by Institutional Review Board of the Children’s Hospital of Chongqing Medical University. The studies were conducted in accordance with the local legislation and institutional requirements. Written informed consent for participation in this study was provided by the participants’ legal guardians/next of kin.

## Author contributions

HL: Conceptualization, Methodology, Writing – original draft, Writing – review & editing. QT: Writing – original draft, Writing – review & editing. KF: Data curation, Investigation, Methodology, Software, Writing – original draft. JC: Data curation, Formal Analysis, Methodology, Writing – review & editing. ZZ: Data curation, Methodology, Validation, Writing – review & editing. SL: Funding acquisition, Resources, Writing – review & editing. LT: Conceptualization, Supervision, Writing – review & editing.
